# Antimicrobial peptides could antagonize uncontrolled inflammation *via* Toll-like 4 receptor

**DOI:** 10.3389/fbioe.2022.1037147

**Published:** 2022-12-07

**Authors:** Danieli F. Buccini, Beatriz C. Roriz, Júlia M. Rodrigues, Octavio L. Franco

**Affiliations:** ^1^ S-Inova Biotech, Programa de Pós-Graduação em Biotecnologia, Universidade Católica Dom Bosco, Campo Grande, Brazil; ^2^ Universidade Estadual do Tocantins, Palmas, Brazil; ^3^ Centro de Análises Proteômicas e Bioquímicas, Pós-Graduação em Ciências Genômicas e Biotecnologia, Universidade Católica de Brasília, Brasília, Brazil

**Keywords:** Toll-like, antimicrobial peptides, myeloid differentiation protein 2, Toll-like 4 receptor, pattern recognition receptors

## Abstract

Antimicrobial peptides are part of the organism’s defense system. They are multifunctional molecules capable of modulating the host’s immune system and recognizing molecules present in pathogens such as lipopolysaccharides (LPSs). LPSs are recognized by molecular patterns associated with pathogens known as Toll-like receptors (TLRs) that protect the organism from pathological microorganisms. TLR4 is responsible for LPS recognition, thus inducing an innate immune response. TLR4 hyperstimulation induces the uncontrolled inflammatory process that is observed in many illnesses, including neurodegenerative, autoimmune and psoriasis). Molecules that act on TLR4 can antagonize the exacerbated inflammatory process. In this context, antimicrobial peptides (AMPs) are promising molecules capable of mediating toll-like receptor signaling. Therefore, here we address the AMPs studied so far with the aim of inhibiting the intense inflammatory process. In addition, we aim to explore some of the interactions between exogenous AMPs and TLR4.

## 1 Introduction

Toll-like receptors are attached to the cell membrane and play a relevant function in the innate immunity (Anwar et al., 2019). They are capable of recognizing lipoproteins, lipopeptides, flagella and nucleic acids from pathogens (Farooq et al., 2021). Once the TLR signaling pathway is activated, a sequence of intracellular events capable of responding to stimuli begins (McClure and Massari, 2014). Humans have 10 TLRs, each performing a response with the help of adapter proteins, which are recruited to mediate the response for each pathogen (O'Neill and Bowie, 2007; Hemmi and Akira, 2005; Khakpour et al., 2015).

Exacerbated activation of Toll-like receptor 4 (TLR4) can lead to hyperstimulation of pro-inflammatory cytokines (Li and Wu, 2021). This intense inflammatory process destroys tissues and can progress to diseases such as rheumatoid arthritis, osteoarthritis, neurodegenerative diseases, autoimmune diseases, Guillain-Barre syndrome, and psoriasis, among others (Cook et al., 2004; Anwar et al., 2019). TLR4 inhibitors can prevent or decrease the exacerbated inflammatory process, contributing to reestablishing tissue homeostasis (Li and Wu, 2021). TLR4 is well characterized in terms of its participation in inflammatory events, and has proved to be a very attractive target for therapies that are antagonistic to the uncontrolled inflammatory process (Slivka et al., 2009; Krishnan et al., 2022).

In this context, AMP are promising molecules capable of mediating the signaling of toll-like receptors, which makes them a therapeutic opportunity for the previously mentioned diseases (Cook et al., 2004; Anwar et al., 2019). AMPs are the organism’s initial line of defense against infections (Hao et al., 2022). These molecules exert various biological functions within the immune response, including cells’ recruitment of monocytes, neutrophils, and immature dendritic cells, inhibition of pro-inflammatory responses, stimulation of cell proliferation, angiogenesis, and wound healing promotion (da Silva and Machado, 2012; Hancock et al., 1995; Zhang et al., 2021).

Many AMPs have been discovered and isolated, and the vast majority have a positive net charge and helical structures (Hao et al., 2022). The liquid cationic characteristic of AMPs attributes an electrostatic attraction to LPS present in the cell wall of Gram-negative bacteria (Sun and Shang, 2015). The adhesion of antimicrobial peptides’ cationic surface to anionic membrane lipids may disrupt membranes and alter cell permeability (Sun and Shang, 2015; Zhang et al., 2021; Krishnan et al., 2022). Furthermore, when AMPs interact with LPSs, they hamper TLR4/LPS binding, blocking the inflammatory pathway activation (Sun and Shang, 2015; Krishnan et al., 2022).

In this context, AMPs seem to be capable of mediating TLR signaling of, being a therapeutic opportunity for related diseases (Cook et al., 2004; Anwar et al., 2019). Therefore, this review aims to search for antimicrobial peptides synthesized with specific activity at the Toll-like 4 receptors.

## 2 Toll-like membrane receptors

Cells need to recognize the imminent danger to the host for innate immune response activation (Duan et al., 2022). This recognition role is played by pattern recognition receptors (PRRs) (Wicherska-Pawłowska et al., 2021). PRRs are able to acknowledge pathogen-associated molecular patterns (PAMPs), damage-associated molecular patterns (DAMPs) and venom-associated molecular patterns (VAMPs) (Wicherska-Pawłowska et al., 2021). PRRs are classified into four groups, including free receptors in serum; membrane-bound phagocytic receptors; membrane-bound signaling receptors and cytoplasmic signaling receptors (Iwasaki and Medzhitov, 2004; Wicherska-Pawłowska et al., 2021; Duan et al., 2022).

Among the PRRs is an evolutionarily conserved family named TLRs (Hua and Hou, 2013). TLRs are found on the membrane surface of macrophages/monocytes, dendritic cells, natural killer cells, mast cells, neutrophils, eosinophils, epithelial cells and T and B cells, all of which are produced from adaptive immune response (Hua and Hou, 2013; McClure and Massari, 2014). Moreover, Toll-like receptors are type I transmembrane glycoproteins; single-pass with an intracellular region, a transmembrane region, and an extracellular region composed of 18–25 leucine-rich repeat copies (LRR) (Kawai and Akira, 2010; Li and Wu, 2021). The multiple LRR sequence forms a horseshoe-shaped protein adaptable to ligand binding and recognition of external (convex) and internal (concave) surfaces (Li and Wu, 2021). Mammalian TLRs are activated when a ligand connection induces them to form dimers or oligomers (Kawai and Akira, 2010; Kawasaki and Kawai, 2014). All mammalian TLR proteins have a TIR (Toll-IL-1 receptor) domain in the cytoplasmic region, which interacts with other TIR-like domains, usually on other signaling molecules (Kawasaki and Kawai, 2014).

TLRs are able to recognize molecules that are released by pathogens under stress and on death (Akira et al., 2006; Farooq et al., 2021). PRRs are attached to intracellular signal transduction pathways that trigger several cellular responses, including the production of inflammation-promoting molecules (pro-inflammatory cytokines) that will recruit other cells (macrophages and neutrophils) that are capable of destroying the microorganisms (Akira et al., 2006).

Ten TLRs were described in humans thus far, classified according to their location (Hemmi and Akira, 2005; Khakpour et al., 2015). The TLRs TLR1, TLR2, TLR4, TLR5, TLR6 and TLR10 are components of the cell surface; they recognize the presence of biomolecules (such as lipids, lipoproteins and proteins) present on the pathogen membrane and induce the inflammatory response (Nagyőszi et al., 2010; Sandig and Bulfone-Paus, 2012). TLR3, TLR7, TLR8 and TLR9 receptors are located in intracellular regions capable of recognizing nucleic acids from viruses and/or microorganisms, inducing type I IFN inflammatory responses (Duan et al., 2022).

Several bacterial infections are recognized by TLR4, due to the receptor’s ability to recognize bacterial LPS (Fan and Malik, 2003). For this recognition, the TLR4 ectodomain uses an accessory protein, MD-2 (myeloid differentiation protein 2—glycoprotein with a sandwich structure of two antiparallel beta sheets) that performs the function of correctly driving TLR4 to the cell surface, thereby recognizing LPSs (Fan and Malik, 2003; Hornef et al., 2003; Anwar et al., 2019; Stolberg-Stolberg et al., 2022). LPSs are detected and captured by LPS-binding proteins present in the blood or extracellular fluids and transferred to a second protein called CD14 (inserted on macrophages, neutrophils, and dendritic cells plasma membrane) (Mitchell et al., 2016; Chen et al., 2019). CD14 is responsible for increasing the sensitivity of the TLR-4-MD-2 signaling complex to find LPSs on the picogram scale (Mitchell et al., 2016; Chen et al., 2019).

After LPS interaction with TLR-4, conformational changes occur in the receptor that bring two adapter proteins closer to domains similar to the Toll/interleukin-1 receptor (TIR) in the intracellular portion (Halabi et al., 2017). The signaling cascade is initiated by four adapter proteins, including MyD88 (myeloid differentiation factor 88), MAL/TIRAP (MyD88-adapter-like/TIR-associated protein), TRIF (Toll-receptor-associated activator of interferon) and TRAM (Toll-receptor-associated molecule) (Yong et al., 2019).

The signaling pathway can be classified into two distinct intracellular classifications: the MyD88-dependent pathway and MyD88-independent pathway, which comprise the TLR3 pathway and the TLR4 pathway (Yong et al., 2019). The MyD88-dependent pathway occurs through association with the MAL protein and start when MyD88 along with IRAK 4 (Interleukin—1—receptor associated kinases) stimulate the activation of IKKs (Serine Kinase) and then phosphorylation of IkB (Kinase IkB), to finally release the NF-kB (Nuclear Factor Kappa-Light-Chain-Enhancer of Activated B Cell) allowing its the translocation to the cell nucleus (Mussbacher et al., 2019; Wang et al., 2019; Yong et al., 2019).

It is worth noting that NF-kB is present in the cell cytoplasm in its inactive form, and after phosphorylation it is translocated to the nucleus, thus inducing gene transcription related to the inflammatory response. The genes responsible for the expression of mediators include TNF-α (tumor necrosis factor), interleukins (IL) 6, IL-1β, interferon gamma (IFN-γ), chemokines, adhesion molecules and antimicrobial peptides (β-defensins, cathelicidins) (Cook et al., 2004; Anwar et al., 2019). Antimicrobial peptides are promising molecules capable of mediating the signaling of toll-like receptors, which makes them a therapeutic opportunity for related diseases (Cook et al., 2004; Anwar et al., 2019).

## 3 Antimicrobial peptides activity on Toll-like receptor 4

Antimicrobial peptides are part of the defense mechanisms of many organisms; they have the most varied activities against microorganisms and also immunomodulate the host’s immune response. AMPs have several characteristics that attribute variability to activities, such as secondary structure (α-helix, β-sheet, and random-coil); amino acid residue composition; three-dimensional structures, molecular masses/charges and hydrophobicity (Peri and Calabrese, 2014; Hao et al., 2022).

Cationic AMPs show attraction for anionic components, such as the plasma membrane of Gram-negative bacteria and LPS (Lei et al., 2019). Cationicity linked to hydrophobicity promotes AMPs’ interaction with fatty acid chains, further favoring LPS binding present in membranes (Hancock et al., 1995; Shanmugam et al., 2012). Thus, AMPs can present mechanisms and structures with varied actions (Peri and Calabrese, 2014), ranging from interactions with molecular charges to the influence of gene expressions (interfering with the beginning of the TLR4 signaling pathway, for example) (Cook et al., 2004; Anwar et al., 2019).

**FIGURE 1:**
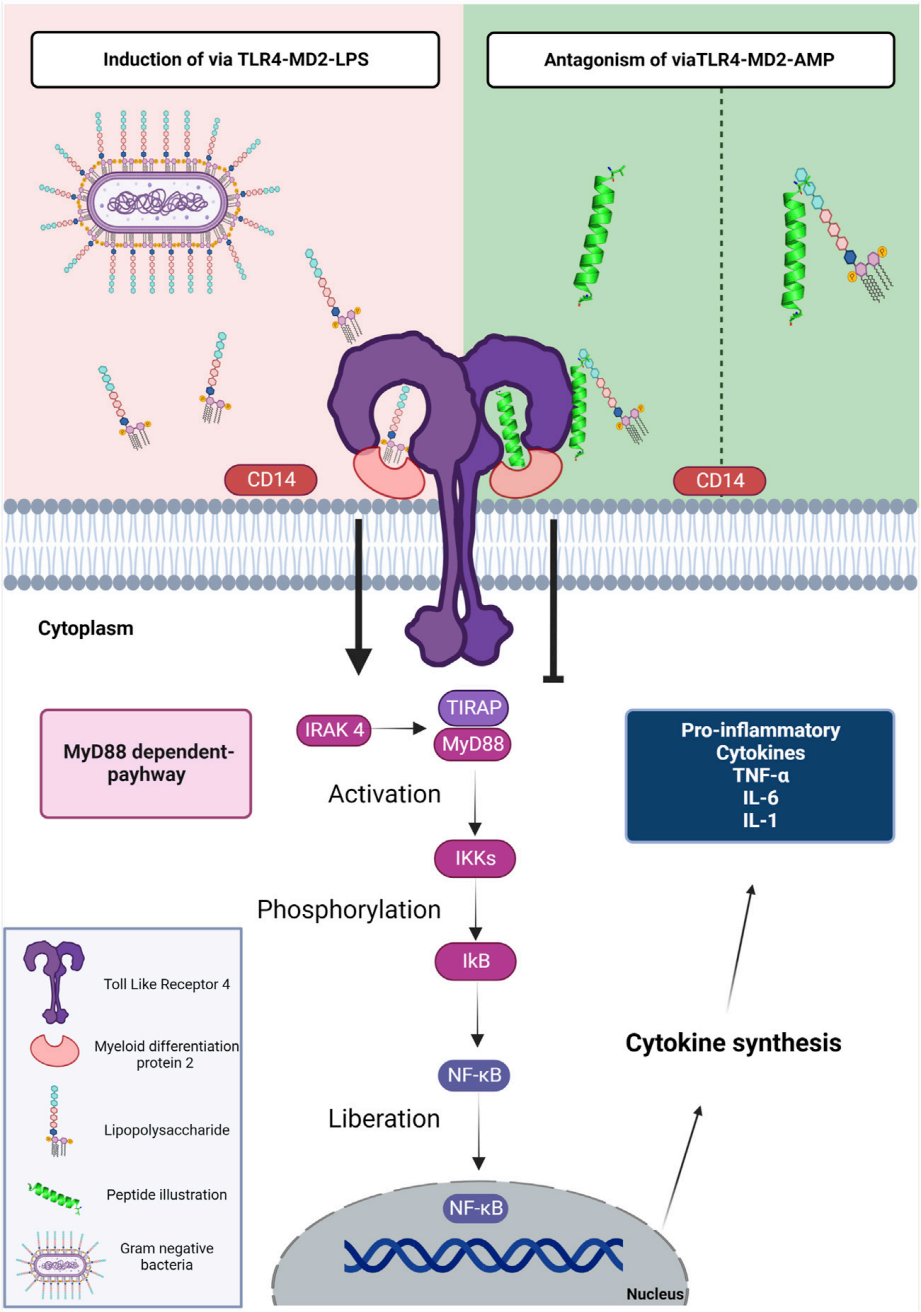
Schematic representation of the mechanism of LPS, AMPs and TLR4. The LPS present in gram-negative bacteria is detected by the CD14 protein. TLR4 uses an accessory protein, MD-2, which performs the function of correctly directing TLR4 to the cell surface, thus recognizing LPS and forming the TLR-4-MD-2 complex (induction *via* TLR4-MD2-LPS). After the interaction of LPS with TLR-4, conformational changes occur in the receptor that leads to the approximation of two adapter proteins in the intracellular portion; TIRAP and MyD88. In this way, the signaling cascade is activated by the MyD88-dependent pathway; this cascate starts when MyD88 along with IRAK 4 stimulate the activation of IKKs and then phosphorylation of IkB, to finally realese the NF-kB. Thus, NF-κB is translocated to the cell nucleus, inducing the transcription of genes associated with the inflammatory response. The genes are responsible for the expression of mediators: TNF-α, IL-6, IL-1β, IFN-γ, chemokines, and adhesion molecules. The TLR4-MD2-AMP pathway can mediate TLR-4 signaling in two ways. The first is interacting with LPS; preventing the encounter of LPS with CD14 and subsequently the TLR-4-MD-2 complex. The second way is the interaction of AMPs with TLR4 which occupies the binding site and prevents the interaction of LPS with the TLR-4-MD-2 complex. The interaction of AMPs with the LPS or TLR-4-MD-2 complex shows antagonistic activity to the MyD88-dependent signaling pathway. Figure created with BioRender.com.

Synthesized AMPs have been an important pharmacological target capable of interfering with the interaction of LPSs and TLR4 (Figure 1) (Peri and Calabrese, 2014). Interactions between the LPS and its receptor, TLR4, with consequent activation of signal transduction pathways, trigger the transcription and secretion of pro-inflammatory cytokines (Shanmugam et al., 2012). TLR4 has been evaluated as the receptor with the largest number of clinical trials with interest as treatment of a variety of pathologies including cancer, viral infection, immune disorders and inflammation (Anwar et al., 2019).

Scientists have sought peptides with TLR4 inhibitory activity acting on MyD88 pathways, both independent or dependent, enabling these inhibitory peptides to turn into therapeutic targets acting in an inflammatory cytokine storm, a characteristic of various pathologies (Lee et al., 2019). Cathelicidins are cationic peptides that were initially portrayed for their antimicrobial and membrane-disruptive activity. Subsequent research elucidated the cathelicidins contact with bacterial membranes or specific membrane components, demonstrating that cathelicidins are attracted to the microorganism membrane *via* electrostatic interactions between negatively charged lipids on the bacterial outer membrane and the peptide positive charge (Scheenstra et al., 2020).

The formation of the complex LPS-cathelicidin plays a substantial role in preventing the binding of the LPS to the TLR4, therefore negatively regulating innate immune responses. Researchers found that the cathelicidin class of peptides inhibits TLR4 activation and alters cell membrane function; the results showed that the synthesized peptides LL-37 (LLGDFFRKSKEKIGKEFKRIVQRIKDFLRNLVPRTES) were able to mediate the anti-inflammatory response *in vivo* and also *in vitro*. That form of cathelicidin peptide named LL-37 acts preventing LPS binding in dentric cell (Di Nardo et al., 2007; Scheenstra et al., 2020). Peptides discussed in this work are summarized in Table 1.

**TABLE 1:** Activity of antimicrobial peptides that act on the Toll Type 4 receptor.

Sequence	Mechanism of action	Assay type	Reference
LL-37	LLGDFFRKSKEKIGKEFRIVQRIKDFLRNLVPRTES	Binding to the TLR-4/MD2 complex and stimulates the release of chemocytes and cytokines	*In vitro* and *in vivo*	Di Nardo et al. (2007)
LTAa	IGKEFKRIVQRIKDFLRNLVPRTEKEKKEVVE	Binding to the TLR-4/MD2 complex and stimulates the release of chemocytes and cytokines. This peptide has been the most favorable to bind to the complex.	*In vitro* and *in vivo*	Zhang et al. (2019)
SPA4	SLQGSIMTVGEKVFSSNGQS	Anti-inflammatory activity, by binding to the TLR4- MD2 complex	*In vitro* and *in vivo*	Awasthi et al. (2021)
Papiliocin	RWKIFKKIEKVGRNVRDGIIKAGPAVAVVGQAATVVK	Competitively inhibits the LPS-TLR4/MD-2 interaction by directly binding to TLR4/MD-2.	*In vitro* and *in vivo*	Krishnan et al. (2022)
CA(1–8)M(1–18)	KWKLFKKIGIGAVLKVLTTGLPALIS-amide	Performs the TLR4 antagonista activity.	*In vitro*	Facchini et al. (2018)

A structure-based of the LL-37 peptide allowed researchers to promote the hybridization of native peptides, a new class of immunomodulatory peptides (Zhang et al., 2019). Hybridization combines the main advantages of peptides with the aim of prolonging the half-life and immunoregulatory activity, in addition to reducing their cytotoxic effects (Zhang et al., 2019). Three hybrid peptides were synthesized by combining a characteristic fragment of LL-37 with a thymosin alpha-1 (Tα1) center, a naturally occurring peptide that has immunomodulatory activity (Zhang et al., 2019). Among them, the LTAa peptide (IGKEFKRIVQRIKDFLRNLVPRTEKEKKEVVE) was the most active peptide, exhibiting immunomodulatory effects since LTAa stimulates the TLR4-NF-kB pathway enhancing MyD88, TLR4 and TRAF6 expression as well IkB-α and NF-kB phosphorylation (Zhang et al., 2019)”.

Protein-A (SP-A) interacts to TLR4 receptor (Awasthi et al., 2011). SP-A is a polypeptide present at lungs surfactant fluid, which, together with phospholipids and lipids, has the function of surface tension decreasing within pulmonary alveoli (Awasthi et al., 2011). SP-A has been shown to have direct interactions with TLR4 suppressing inflammatory cytokines response (Awasthi et al., 2011). Subsequent to this study, seven peptides derived from C-termini CRD region of human SP-A were synthesized. Among the seven peptides synthesized, SPA4 strongly bound to TLR4-MD2, inhibiting TNF‐α release when dendritic cells were stimulated with LPS (Awasthi et al., 2011). By using in vitro and in vivo assays, SPA4 peptide has a mechanistic role of amino acids D-2 and NYTXXXRG, responsible for anti-inflammatory activity by binding to the TLR4-MD2 complex (Awasthi et al., 2021). Furthermore, the central 6-14 amino acids residues “SDGTPVNYT” of SPA4 peptide form a turn, and amino acids on either side (GDFRY and NWYRGE) form flexible arms that lead SPA4 peptide for binding to TLR4 (Awasthi et al., 2015). By using molecular modeling studies, distances predicted amino acids (D, N, Y, T, R, G) of SPA4 peptide and (E, Q, E, K, F) of TLR4 7 ranged between 3‐10 Å (Awasthi et al., 2015).

Another class of AMPs with potent action on toll-like receptors is that of the cecropins (Hoffmann, 1995; Wu et al., 2018). This peptide was identified more than 40 years ago in conjunction with a broad discovery of other insect-derived AMPs (Hoffmann, 1995; Silvestro et al., 2000; Wu et al., 2018). These molecules are widely studied not only for their physiological role in insect immunity, but also for their immunomodulatory characteristics (Hoffmann, 1995; Silvestro et al., 2000; Wu et al., 2018).

Krishnan et al., (Krishnan et al., 2022), reported a new molecular mechanism with papiliocin antagonistic effect mediated by its direct binding to the TLR4/MD-2 complex. Such antagonistic effect occurs once peptide competes with LPS for TLR4/MD-2 complex binding site. Due to this antagonistic effect, papiliocin was capable of inhibit the inflammatory cascade caused by TLR4 activation and blocking the p-NF-κB nuclear translocation, resulting in nitric oxide and TNF-α production inhibition RAW 264.7 cells stimulated with LPSs (Krishnan et al., 2022).

The combination of peptides with other classes of molecules is also of pharmacological interest, since synergism between two compounds can result in inactivation of the TLR4 receptor. These results were clarified by researchers that surveyed the combined action between the anionic glycolipid FP7 (diphosphorylated glucosamine monosaccharide) and cationic peptides, reporting that it can boost the activity of AMPs of the cecropin class (Facchini et al., 2018). The results showed that cationic peptides can increase the activity of cecropin AMPs (cecropin A melittin). In synergism with FP7, peptides CA(1–8)M(1–18) (KWKLFKKIGIGAVLKVLTTGLPALIS-amide) and LL-37 (LLGDFFRKSKEKIGKEFKRIVQRIKDFLRNLVPRTES) were the most effective. Multiple possible mechanisms of action may be related to the direct binding of AMPs to CD14 and MD-2 receptors; Moreover FP7 aggregation state of is an allosteric action of AMPs that can balance F27-MD-2-TLR4 deactivation (Facchini et al., 2018).

Despite the great progress achieved in the interaction between PRRs and ligands, finding and identifying mechanisms of action that are involved in ligand recognition has been a challenge (Li and Wu, 2021). Compared to small molecules and chemical compounds, peptides have better efficacy, safety, selectivity and potency, which makes peptides attractive as future therapeutic targets. However, the number of antagonist peptides that are targeted by TLRs is still lower when compared to other antagonists, such as chemical agents and antibodies (Lee et al., 2019).

## 4 Future prospects

Despite advances in PRR studies, little research has focused on discovering the AMPs’ mechanism of action targeting TLR4. In that view, peptides here reported present the LPS binding capability and further evidence of peptides’ ability to interact with TLR4. In that case, AMPs bind to TLR4, competing with LPS for the same receptor (Krishnan et al., 2022). In summary, AMPs here described may antagonize the inflammatory signaling cascade *via* TLR4 and, as a consequence, prevent the expression of pro-inflammatory cytokines *via* NF-ḳB.

Nevertheless, only a few studies have showed AMPs specifically interacting with TLR4. Among them, data scarcity that address AMPs and TLR4 interactions is clear and novel approaches for better understand such interaction must be performed.In addition to a better understanding of mechanism of action, it is vital to search for molecules that antagonize the uncontrolled inflammatory process, given the number of diseases that are aggravated in this process, such as rheumatoid arthritis, osteoarthritis, neurodegenerative diseases, autoimmune diseases, Guillain-Barre syndrome and psoriasis (Anwar et al., 2019; Cook et al., 2004).

A gold tool that could help and further contributes to this process consist in OMICs tools associated to bioinformatics. In that view a global problem visualization could help to drive the design and further synthesize specific peptides for desired receptors. An improved specificity of these molecules may be the key for treatment of severe autoimmune diseases that present an exacerbated inflammatory process.
